# Popular Tag Recommendation by Neural Network in Social Media

**DOI:** 10.1155/2023/4300408

**Published:** 2023-05-29

**Authors:** Mohammad Jafari Sadr, Seyedeh Leili Mirtaheri, Sergio Greco, Keivan Borna

**Affiliations:** ^1^Department of Computer Science, Faculty of Mathematical Sciences and Computer, Kharazmi University, Tehran, Iran; ^2^Department of Electrical and Computer Engineering, Faculty of Engineering, Kharazmi University, Tehran, Iran; ^3^Department of Informatics, Modeling, Electronics and System Engineering, University of Calabria, Calabria, Italy

## Abstract

Although “a picture is worth a thousand words,” this may not be enough to get your post seen on social media. This study's main objective was to determine the best ways to characterize a photo in terms of viral marketing and public appealing. We have to obtain this dataset for this reason from the social media site such as Instagram. A total of 1.4 million hashtags were used in the 570,000 photos that we crawled. Prior to training the text generation module to produce such popular hashtags, we had to determine the components and features of the photo. We trained a multilabel image classification module using a ResNet neural network model for the first section. In order to create hashtags pertaining to their popularity, we trained a cutting-edge GPT-2 language model for the second portion. This work differs from others in that, and it initially offered a cutting-edge GPT-2 model for hashtag generation using a combination of the multilabel image classification module. The popularity issues and ways to make an Instagram post popular are also highlighted in our essay. Social science and marketing research can both be conducted on this subject. Which content can be considered popular from the perspective of consumers can be researched in the social science setting. As a marketing strategy, end users can help by offering such well-liked hashtags for social media accounts. This essay adds to the body of knowledge by demonstrating the two possible uses of popularity. Compared to the base model, our popular hashtag generating algorithm creates 11% more relevant, acceptable, and trending hashtags, according to the evaluation that was carried out.

## 1. Introduction

In social networks, the ability to automatically tag photographs with a hashtag and follow it has grown in significance [1]. Given the ability to follow hashtags (tags) on social media sites like Instagram and their significance in boosting visitors and recommending hashtags based on the components of an image is effective.

Hashtag recommendation first is distinct into three categories: textual (for example a social network like Twitter), visual data [2, 3] (for example Instagram social media), and multimodal (a combination of textual and visual data) [4, 5].

There are lots of ways for hashtag recommendation in both categories from old methods such as similarity measures [6–9] and classical machine learning methods [10–12] or newer models such as topic models [13–16], topical translation models [17–21], and Deep Learning models [4, 22–27]. Some research studies [3–5] work both on text and image data together. These works rely on the linkage of visual and textual data. This study [4] proposed the coattention LSTM model to generate tags, and this article presented a multimodal model for video tagging and also proposed the LSTM neural network for image captioning based on videos thumbnails [5]; however, that is not the subject of our study. Most of them are about Twitter social network and textual data [28].

In this study, specific hashtags are suggested in order to boost the number of views of the image or content, taking into account the popularity of each photo and hashtag on social media. These tags can be created using conventional image tagging and diverse image annotation, respectively [29–31].

Multilabel classification algorithms have been utilized in our implementation to detect image components and extract image attributes in the first phase, which will result in the generation of these tags or hashtags. In the second stage, tags have been generated using transformers text generations models based on the level of popularity. The end result is the creation of trending hashtags and estimation of visitors.

One of the innovative parts of this research is how it leverages transformers to generate hashtags for photographs in accordance with the popularity of hashtags. By utilizing the most recent cutting-edge models, we attempted to supply the best method for tagging photographs depending on popularity in this study. We will teach image classification techniques such as multilabel algorithms and text creation techniques in accordance with the primary goal of this study. [32–35]. Multilabel classification methods are among the new methods in the field of computer vision that can be conducted using different techniques and methods [29, 30, 36–38]. Examples of these models will be introduced in the next sections. Due to the generation and recommendation of popular hashtags in this study, it was necessary to study the methods of text generation.

This study continues as follows: in Section 2, we review some methods that cover in our article.  Section 3 discusses related works, and in Section 4, we present our method for image tag recommendation. In Section 5, we conduct some experiments and analyze our implementation compared to other methods, and in Section 6, we conclude our article and suggest some future works.

## 2. Literature Review

In the following, we looked at some of the subjects we covered in our study. First, we looked at techniques for classifying images with multiple labels, and then, we looked at models for generating natural language.

### 2.1. Image Classification

Among various multilabel image classification methods, such as machine learning algorithms (supervised methods), support vectors machines, and artificial neural networks are the most popular. These methods include the nearest neighbor algorithm [38], multilabel decision tree [36], and ranked support vector machine [37]. Lack of recognition of correlations within labels, not working with too many labels, and the need to prune trees are some of these methods' problems. Methods such as convolutional neural networks tried to solve previous problems using Deep Learning [39, 40]. In these methods, classification models are divided into four categories:

- Transfer Learning: based on the structure of convolutional neural networks and using training and rearrangement on multilabel images, the method tries to recognize different labels due to the high quality of these models in single-label images. This work is conducted by changing the last layers of these networks for multilabel image classification.
- Multilabel Image Segmentation: in this method, according to the placement of objects in the image and recognizing the area of each object in the image, different objects are detected. Due to these models' complexity in identifying boundary areas, they can be called high computational methods.
- Extraction of correlated features: based on selecting the correlation between different labels and the relationship between them, this method tries to identify the elements in the image, which has its complexity in aggregation with convolutional neural networks.
- Ensembling Technique: in this method, based on the aggregation of different models of convolutional neural networks and calculating the average output of these various networks, elements of images are detected, and due to the high accuracy of convolutional neural network models, a significant outcome of this method can be expected.

### 2.2. Natural Language Generation

In accordance with the definitions of natural language processing, natural language generation refers to the production of meaningful phrases and sentences using natural language. It is theoretically possible to automatically explain, characterize, and summarize structured inputs by utilizing this technique [41].

However, although being able to generate sentences and phrases, this approach is unable to comprehend these sentences and phrases. Natural language understanding, another component, is needed to comprehend phrases and sentences. It is possible to comprehend human language by employing natural language processing. Expressions that lack structure can be transformed into those that conduct using natural language processing (computer understandable) [42].

In other words, natural language generation and understanding are broad categories of natural language processing that include the interpretation or creation of human language, both in written and spoken forms [41].

In order to understand natural language, one must first determine the subject and entities of the input data (human language) based on the grammar or context of that data.

Natural language processing, which transforms text into structured phrases, and natural language generation, which produces text based on structured phrases [41].

Natural language generation models replicate human language and adjust to writing style, tone, structure, and context using a variety of techniques and algorithms.

The use of a template or dynamic document production is two fundamental methods for NLG. Despite being the main method for NLG, the second approach has come a long way from simple patterns to more sophisticated techniques. Various strategies have been put forth in the interim; they broadened the application and improved language creation capability [41]. In the following, we will discuss some of these approaches:

- Simple Filling the Gap: this strategy is one of the oldest ones. The amount of information required to finish texts with a predetermined structure is minimal. This method uses data that are retrieved from a spreadsheet and database table row to automatically fill in the blanks. Natural language generation does not typically use this simple methodology because it can only alter certain portions of the text in practice.
- Scripts or Text Generation Rules: using general programming methods such as scripts and professional rules, simple filling the gap methods were developed. Using web templating language and embedding templates within scripting language can be conducted to define complex words, loops, and access code libraries. The professional approach has a similar function to the previous approach but uses professional rules instead of scripts. Although this approach was more potent than the simple filling the gap method, it lacked language skills in generating complex, high-quality texts.
- Grammatical operations at the word level: with the logical advancement of pattern-based approaches, grammatical operations at the word level were added. These features make it easy to incorporate text creation tools such as punctuation, phonology, word spelling, and exception control. It was simpler to produce sentences with proper syntax and intricate patterns while using these functions.
- Dynamic sentence Generation: finally, we can generate sentences dynamically by switching from pattern-based approaches to the dynamic language creation methodology. This method allows for the dynamic generation of sentences based on the representation of predicted linguistic structure or meaning. Dynamic generation refers to the ability of the system to reliably construct sentences in the majority of circumstances without the developer having to specify boundary constraints. We will also be able to optimize sentences in this system using a variety of techniques, including reference, aggregation, ordering, and conjunctive.
- Dynamic Document Generation: the macrolevel of dynamic sentence production can result in a text that is helpful and relevant to readers, as well as being well-structured as a narrative. The function of this strategy relies on the text's intended audience. Consider using a model of reasoning and behavior change, mimicking human speech, or summarizing business intelligence data based on an examination of important business KPIs.

Several distinct algorithms are used for natural language generation. The following methods have been suggested to address the issue of text construction in natural language, which has always been a challenge. [41].

- Markov Chain: the hidden Markov chain model is one of the first algorithms for natural language generation [43]. This model tries to predict the next word in the sentence by using the current word and calculating each unique word's probability as the next word. It was previously seen in early versions of smartphone keyboards, where suggestions for the next word in the sentence were made.
- Recurrent Neural Network: in general, neural networks are used as models to simulate how the human brain functions. Each portion of a series is passed through a forward network in recurrent neural networks, and the output of each part of these networks is regarded as the input for the following section in the sequence. The training algorithm estimates the probability of the following word and stores the previous word model it encountered in memory before repeating. The model determines a probability for each word in the dictionary based on the word before it. A term with the highest probability is then chosen by the neural network and stored in the model memory. Recurrent neural networks have become a perfect model for memory since they could retain conversational situations. Therefore, only the most recent phrases in the series can be used to forecast the prediction of the following terms. This issue prevents recurrent neural networks from producing cohesive phrases with a long succession [44].
- Long Short-Term Memory: To solve the problem of long sequences, a new architecture of the recurrent neural network called long short-term memory (LSTM) was proposed [45]. This architecture has four layers as opposed to the recurrent neural network's two layers. These four layers—a unit, an input unit, an output unit, and a forget unit—enable the recurrent neural network to modify the frequency of reminders or forgetting dependent on the volume of information flowing through the unit at any one time. The forgotten unit disregards its most recent knowledge once a sentence ends because it understands that the topic might change. The network can track just useful information precisely by utilizing this. Additionally, this architecture addressed the issue of the gradient's abrupt development, which causes issues during the training of recurrent neural networks. After all these advancements, the model eventually discovered the capacity for processing and analysing.
- Transformers: The transformer was first introduced in reference [33]. A new method is also called the self-attention mechanism. Transformers consist of sets of encoder stacks for processing any input and sets of decoder stacks for generating sentences as output. Compared to long short-term memory architecture, transformers operate in only a few short steps. The self-attention mechanism directly simulates the relationship between all the words in a sentence. Unlike the long short-term memory model, the transformer uses the representation of all words according to their context, without having to compress all the information into a given length, thus allowing the system to generate longer sentences without the need for managing heavier calculations. One practical example of transformers for language generation is the OpenAI GPT2 language model [34]. The model learns to predict the next word in a sentence by focusing on words that are already seen in the model or are related to the next word. One of the newest models offered by Google Research is transformers with a two-way encoder representation called BERT [35]. This langauge model has shown very great results for various applications of natural language processing.

### 2.3. Popularity in Social Network and Image Hashtags

The visual content of the photo, the textual information linked with it (such as hashtags, which can be found by searching for keywords on any social network), and the popularity of the photo's creator are the three primary factors in determining an image's popularity [46].

A photo's ability to be seen more widely depends on the presence of textual information alongside the image. One of the elements that increase the number of photographs viewed is the number of hashtags assigned to the images, as well as how straightforward these hashtags are. The image's views will increase because the straightforward hashtags are simpler to search for or find [46].

A hashtag with the # symbol is a prefix symbol, and one of the metadata tags used in social networking and microblogging services. If we want to define a hashtag in simple, it is a tag that is used to categorize and share posts and comments on a specific topic globally and beyond the friends' list. The hashtag provides a tool for classifying such content so that people can search for that hashtag to access a collection of content that includes that hashtag. They usually associate the most keyword related to that topic with a hashtag. This pairing is carried out using the # sign before the word. You can use allowed letters, numbers, and symbols in hashtag registration, but symbols such as $ or % are not allowed, and you are not allowed to register. The hashtag was first created by Twitter and has since been used by many social networks, including Google Plus, Facebook, Flickr, Instagram, Friend Feed, YouTube, Pinterest, and Telegram.

Popularity is difficult to gauge because each social network has different standards. Instagram, for instance, gauges popularity by counting comments or likes. A reliable indicator of popularity is the number of likes. Though it shouldnot be the only one taken into account. To put it another way, something becomes well-known when many of people notice it. This criterion is known as board; no platform specifies a measurement known as a hashtag range. Knowing the board of hashtags might be useful when hashtags are used as references to already existing material [47].

The hashtag on Instagram is nothing but a single word (it may be a few words, but due to the implemented structure, it is considered as one word) with a hash sign (#) in the subject of each post or inserted in the comments section of the content.

One of the uses of hashtags is to increase the view of images, and as a result, it helps to increase the popularity of the photo or content [48].

## 3. Related Work

Based on the review of survey [28], hashtag recommendations on social network systems are categorized into five sections. These categories are methods based on similarity measures [6–9] and classification models based on old-fashioned machine learning methods [10, 12] and models based on topic modeling [13–16], topical translation models [17–21], and Deep Learning models [4, 22–27]. Much of the research studies are conducted on Twitter social networks and only about textual content. But also, there is research on mixed texture and visual data [3, 4]. This hashtag recommendation system is based on CNN and LSTM models and works with a mix of other types of data like images. Also, based on [28], popularity prediction in the hashtag recommendation field is not researched as much as others. Most of this research also conducted on textual information and Twitter social network [24, 49].

Among the research examining the popularity of hashtags is HARRISON Dataset [50]. In this dataset, focusing on 50 popular hashtags, they tried to build a basic model using convolutional neural networks that could act as the multilabel classification model. Among the problems of this research is the lack of a widespread understanding of visual information, not the use of dependencies between different classes of labels and the misunderstanding of textual information [50].

To compare the method of generating hashtags, this research [51] recommends hashtags for Instagram to focus on one-step learning and compare it to supervised methods. But, in this research, there is no analysis of its Instagram hashtags.

Another research works on text and image data [3]. They used a multimodal neural network that consists of an encoder for feature extraction and a decoder for the recommendation. But, in this research, popularity is not considered for hashtag generation.

Also, we study and review the method presented in this study [32], which used the convolutional neural network and characters embedding in recurrent neural networks (LSTM) method to generate hashtags. Therefore, by redeveloping this method, training the network presented with our study's data, and using it as a base model, we compare the quality of generated hashtags and examine the popularity of the generated hashtags of our research.

According to the research and data, there have only been a few works on hashtag generation. There is no research on the generation of hashtags depending on popularity. According to a review of publications on popularity and image classification, the generation of hashtags and image recognition are two of the newest and most well-liked topics in the field of computer vision.

## 4. The Proposed Method

For the proposed method, it was necessary to identify image labels according to Instagram hashtags. For this purpose, we developed a multilabel classification algorithm by Deep Learning methods. We have conducted that by fine-tuning Resnet-50 Network for multilabel image classification and feature extraction to generate popular hashtags. We developed the natural language generation model based on transformers and OpenAI GPT-2 [34] language model. We use features extracted from the previous part to train the transformer network and generate popular hashtags with attention to the input data (images). Finally, according to the evaluation metrics, we generate hashtags with an estimated number of image (post) visibility. As follows, we explain each part in detail.

### 4.1. Multi-Label Image Classification

Using the FastAI library [52] a training algorithm for Resnet50 [53] with multilabel classification capability has been developed. We will use this algorithm to build a multilabel image classification model. For the training method, we use a spreadsheet file where in the first column there is the path of each image file, and in the second column, each image hashtag was prepared. Each image file was read from its way was processed as a data frame, and hashtags were identified as labels for each image file. By dividing the number of images and the number of hashtags, every image by average was tagged with 2.46 hashtags. As the number of unique hashtags is too large for the training set of multilabel image classification module, and due to the sparsity of the hashtags matrix, first we examine the hashtags with NLP preprocessing functions such as misspellings, stemming, and removing unnecessary characters with NLTK [54]. After this process, we got 1031 tags that describe images the most, and we set these numbers of tags for the training set.

In the first phase, to Fine-Tune the Resnet50 Neural Network for a multilabel technique, it is necessary to divide the data into two categories: training and validation, for which 80% of the data are considered as training and 20% as validation data in a completely random consideration. In the next step, it was necessary to resize all images' resolution to the specified size, and the same size (as a square) that the selected resolution size in the first phase was 128 × 128, and for this purpose, we used the transformation function of the FastAI library. Also, normalization was performed based on the results of the ImageNet [51], and it should be noted that we considered 64 as a batch size according to the hardware limitations.

After preparing the training and validation data, the training parameters of the convolutional neural network are determined. Also, for the training metric due to the multilabel dataset, we used two metrics: accuracy threshold [55] and *F*-beta score [56].

In the accuracy threshold formula, the predicted values are compared with the real values. After applying the Sigmoid function to the predicted values and comparing it with the threshold, which is 0.5, and the number of valid predicted data is determined according to the target data. This metric is considered for multilabel problems [55].

The f-beta metric (We selected beta = 2 because we try to emphasize the false positive instead of the false negative during training in the multilabel classification part. The multilabel classification module is a primary part of element detection during popular hashtag generation, so we want to be near accurate rather than fallacious.) is the mean between the accuracy parameter and the recall parameter, and its equation is as follows:

(1) $$\begin{array}{c} F_{\beta }=\left(1+\beta^{2}\right)\cdot \frac{precision\cdot recall}{\left(\beta^{2}\cdot precision\right)+recall}\text{.} \end{array}$$

This metric is also used to evaluate multilabel problems [56]. We also considered the threshold for this metric (1) to be 0.5.

**Figure 1:**
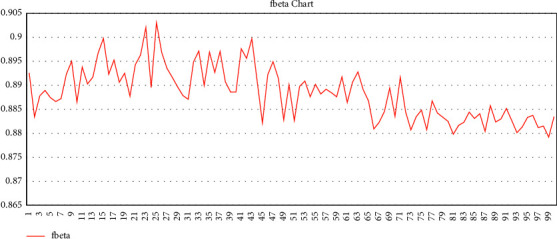
*F*_beta_ measure of last training cycle.

**Figure 2:**
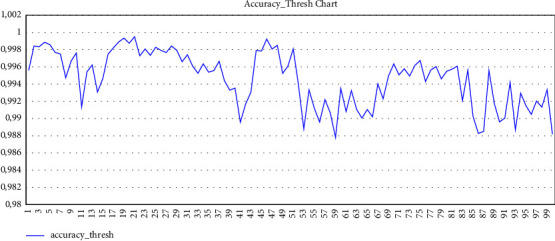
Accuracy threshold of last training cycle.

Determining the learning rate is one of the most important hyper parameters in neural network training [57]. Because of that, we used the cyclic learning rate method. We considered four cycles to train this module, and before each cycle, the learning rate was selected based on the previously mentioned method. After that, a training cycle with 150 epochs was set for training the first cycle. The learning rate was selected for the second cycle, based on the cyclic learning rate method, and we considered 100 epochs for this cycle. For the third cycle, to increase the resolution of the images, we considered 256 × 256 size for the transformation of input images and set the batch size as 16. The third cycle and the fourth cycle, like the previous cycle, were performed with 100 epochs. The graph of the last training cycle based on the metrics is as follows: Figures 1 and 2 (finally, despite the hardware limitations, we achieved the following accuracy according to the evaluation metrics).

This multilabel classification module was used to get the initial labels for the image in the following part (Text Generation Module with attention). The training method for creating hashtags, on which this study is built, will be described in the part that follows.

### 4.2. Popular Hashtags Generation

To generate popular hashtags for the labels extracted from the image, we used one of the state-of-the-art neural network architectures called transformer [33]. For this purpose, we used the library of transformers [58] to train the transformers language module on popular hashtags. To work with this library, we also used one of the latest language models called OpenAI GPT-2 [34], which has achieved amazing results in natural language understanding and natural language generation. In the following, we will explain the details of transformer neural network training.

In order to create trending hashtags, the model first needed to be trained with all of the hashtags relating to each image. In order to train text generation with attention, modifications were performed to the hashtag column data (included in the spreadsheet). The list of hashtags for this part's training set is evaluated by the widely used evaluation function first, and the hashtag combinations with the highest scores are then chosen as the training set. In the next step, by adding the phrase (HASHTAG:) at the beginning and adding the phrase (<|endoftext|>) at the end, we prepared data for model training.

In this stage, we established the network parameters after preparing the network input data. The batch size was set to 64, the number of epochs was 5, the steps to adjust the learning rate were 5000 for each iteration, the initial learning rate was 0.00003, and the maximum length of the sequence was 500, according to the studies and testing that were conducted. In order to minimize the learning rate linearly, Adam's optimization with continuous weight reduction and linear programming by figuring out how many steps to take were also utilized.

**Figure 3:**
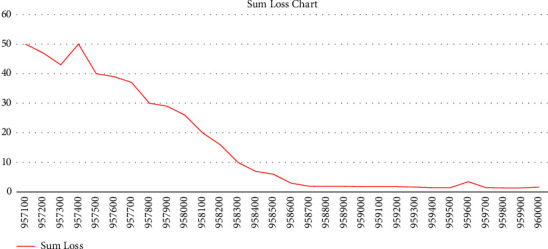
Accuracy threshold.

In the next step, the model was put into the training mode. It was necessary to vectorize the input data, in which we used the GPT-2 tokenization, to input the data for model training. Input phrases are converted to numeric symbols for model training, by using this tokenizer. In the training algorithm, it was tried to consider the maximum input data sequence for model training compared to the maximum sequence length. Each time the data are entered into the model for training, and the model accuracy and loss which is determined using the maximum likelihood estimation function on the model input and output data and backpropagating it into the network. In Figure 3, the last 30 iterations have been reported as one hundred periodic.

To generate popular hashtags, we set another evaluation metric for the model training algorithm that compares the distance of generated hashtags with the list of 100 popular hashtags last updated on February 20, 2020. This evaluation metric is called BLEU [59]. This metric measures the distance between the words in the generated text set compared to the reference text dataset evaluates the generated text and assigns a higher score to consecutive words. This evaluation metric is similar to the recall evaluation metric, so we used this evaluation's average to compare the distance of generated hashtags from popular hashtags. The results of this evaluation will be reviewed and presented in detail in the experiment section.

Finally, as in the previous section, all model parameters are entirely stored and used in the experiment section to evaluate the generated data.

**Figure 4:**
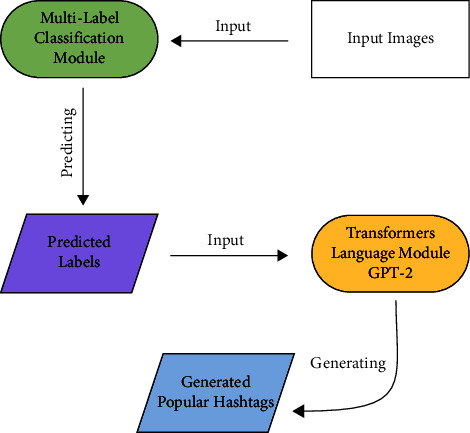
The process of generating popular hashtags of the input image.

Figure 4 shows all parts of the model for generating popular hashtags can be seen. This figure shows the workflow of how popular hashtags are generated according to the content of the input image. First, multilabel classification module predicts the root labels and in the next part, transformers language module generated the list of popular hashtags.

## 5. Experiment

### 5.1. Data

To conduct research on the popularity and also to generate hashtags according to the elements of the image, it was necessary to collect data based on the mentions. Based on this, the social media, Instagram was selected and why Instagram was chosen, the following notes are considered:

- Ability to follow hashtags on Instagram
- Possibility to check the number of likes and comments of each post (image)
- Possibility to access different information according to the selection of hashtags

For this purpose, we study the existing datasets in this regard. According to our study, this article [60] collected data concerning the number of likes and comments of a photography competition called the weekend hashtag project (WHP). In the mentioned dataset, the photos were anonymously saved (a photo file was not available, and only a counter of each photo was available), and users' network information, as well as the number of likes and comments, were available. The original image files were not available in this dataset. For this reason, by taking the idea of this article [60], we selected 72 hashtags related to the mentioned competition, and based on that, we designed an algorithm to collect data according to these hashtags, and all the posts related to these 72 hashtags have been crawled from the Instagram and each hashtag saved in different folders. Also, the information of each post such as textual data, the number of likes, and the number of comments were saved in the format of a text file alongside each image file. The statistics of the total number of collected data are listed in Table 1. The data used to support the findings of this study are available from the corresponding author upon request.

Some of crawled hashtags are such as #whpumbrellas, #whpaquarium, #whpstoryinmotion, #whpmovingphotos, #whpbirdsonawire, #whppetportraits, #whpresolutions, #whpexplore, #whpdoortodoor, #whpsolocolor, #whpstoryportrait, #whpgreatheights, #whpmylibrary, #whplookingup, #whpgreatheights, #whpumbrellas, #whpaquarium, #whpstoryinmotion, #whpmovingphotos, #whpbirdsonawire, #whppetportraits, #whpresolutions, #whpexplore, #whpdoortodoor, #whpsolocolor, #whpstoryportrait, #whpgreatheights, #whpmylibrary, #whplookingup, and #whpgreatheights.

**Figure 5:**
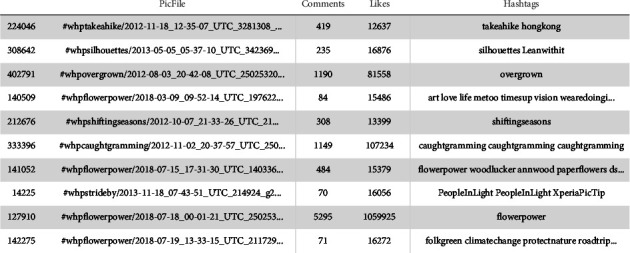
Sample of collected data spreadsheet.

In order to create the dataset for further processing, it was essential to create a spreadsheet for the acquired data. Python's Pandas data frame was utilized for this. The list of all text files was first chosen, and the data were then preprocessed. Each text file's accompanying image files were recognized, allowing the post title and other details to be displayed next to the photographs. The content of the text files was then automatically verified using regular expressions after removing all unnecessary and useless characters. Along with the number of likes and comments, the hashtags used for each post were extracted, and a file matching to each photo's file was saved. The assembled spreadsheet is displayed in Figure 5.

### 5.2. Hardware Specifications and Limitations

For the training and evaluation part, we have used PyTorch v1.2.0 framework and the server with the following properties:

- CPU: Core i7-7700@3.60 GHz
- GPU: GP104 [GeForce GTX 1080]
- RAM: 64 GB@2133 MHz

The model occasionally displays low accuracy in a test mode as a result of the high volume of data gathering, numerous distinct hashtags (about 133,000), and hardware limitations (GPU memory limit—8 GB). A better model can be developed and may ultimately differ slightly from the findings of this study by identifying the best hardware and batch size.

The multilabel model's training is also time-consuming, and lower image sizes must be chosen because of hardware restrictions. Different test modes might arise from selecting photographs with a greater resolution.

Due to the huge volume of tokenization in the implemented algorithm, using better hardware, choosing the batch size, and altering the number of iterations can increase the model's accuracy in producing appropriate hashtags while training the next generation portion. Additionally, the model's randomness in selecting the subsequent words in the text creation method could cause the test data results to diverge.

### 5.3. Evaluation Results

To generate popular hashtags, we used the BLEU evaluation metric [59] to assess the generated hashtags' popularity rate. This evaluation metric is only able to measure lexical diversity. It lacks the ability to measure semantic and syntactic variations [61]. Therefore, a new evaluation metric called BLEURT was introduced by Google Research [61]. Using this evaluation metric, we can examine the generated text (hashtags) according to the semantic changes. It was necessary to compare them with a dataset of reference texts to explore the sample of generated hashtags. We considered the real hashtags used in the image post entered by the real user as the reference text. We randomly selected two test data samples with 800 and 1200 post images from the list of all hashtags in the dataset to evaluate the model presented in this study.

To evaluate this research, we considered the text generation method using convolutional neural network and characters embedding in recurrent neural networks (LSTM) [32] base model method. Some state-of-the-art models [4, 5] as mentioned in our article are not comparable to the output of this study due to the differences between the subjects. We selected this LSTM Model because is the only model related to the output of our study. Then, we examined the base model with the method presented in this study, both in terms of lexical diversity and semantics. Lexical diversity can be measured using BLEU. Also, to evaluate semantic changes, the metric BLEURT was used.

**Figure 6:**
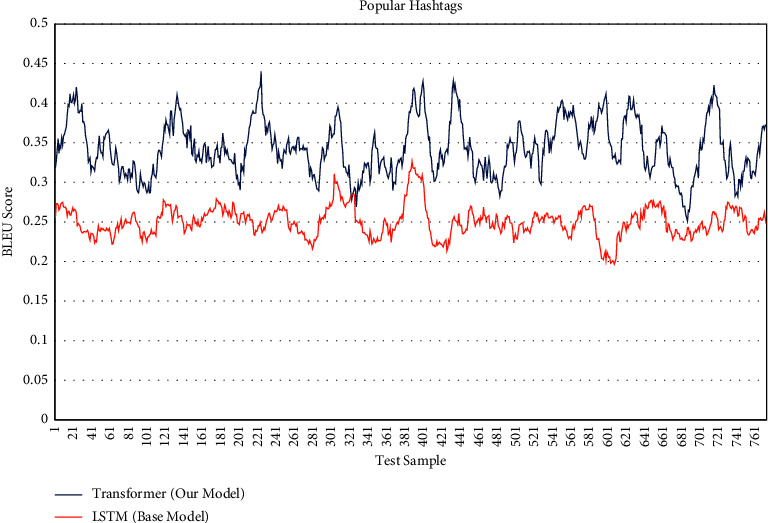
BLEU score of popular hashtags (800 test sample).

We show the difference between generating popular hashtags compared to the base model and the model of this research.  Figure 6 shows BLEU score for each test sample containing 800 images, as can be seen overall for each sample significantly generated more popular hashtags.  Table 2 shows on average that there has been a significant increase in the generation of popular hashtags using the method presented in this study.

**Figure 7:**
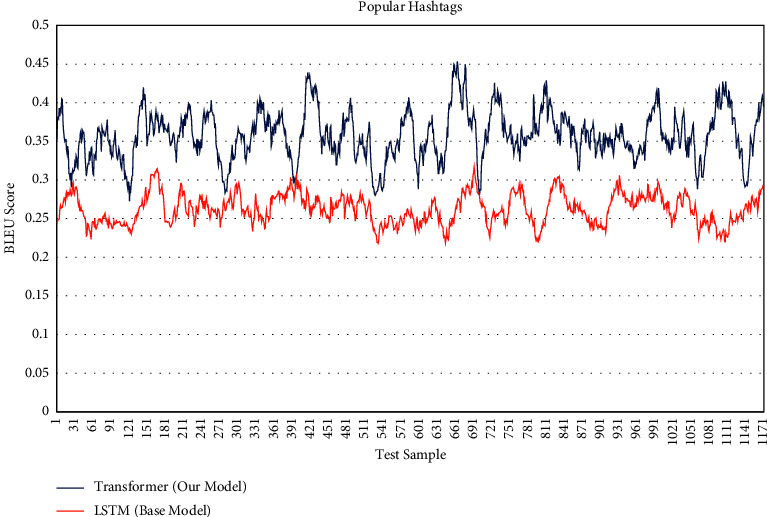
BLEU score of popular hashtags (1200 test sample).

To further confirm the results of this research's implementation, we examined the hashtags generated on other random samples that included 1,200 images. In this re-evaluation, Figure 7 shows BLEU Score of popular hashtags for each 1,200 test sample. As shown in Table 3, there is a significant increase in the generation of popular hashtags.

**Figure 8:**
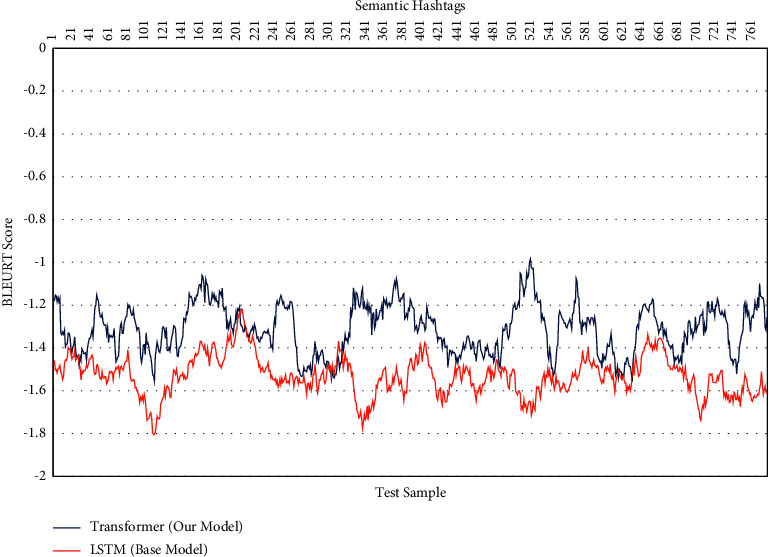
BLEURT score of semantic in generated hashtags (800 test sample).

Only the linguistic variations of the popular hashtags in the sample of generated hashtags were evaluated in the previous sections. We will assess the semantic changes and the significance of the generated hashtags in the following section. BLEURT was utilized for this evaluation.  Figure 8 shows the semantic changes of 800 test samples for generating hashtags in our model and base model. As shown, all test samples are negative based on just examining the semantics of generated hashtags and not syntactically based on the structure of hashtags themselves.  Table 4 shows the average accuracy obtained for a sample of 800 data.

**Figure 9:**
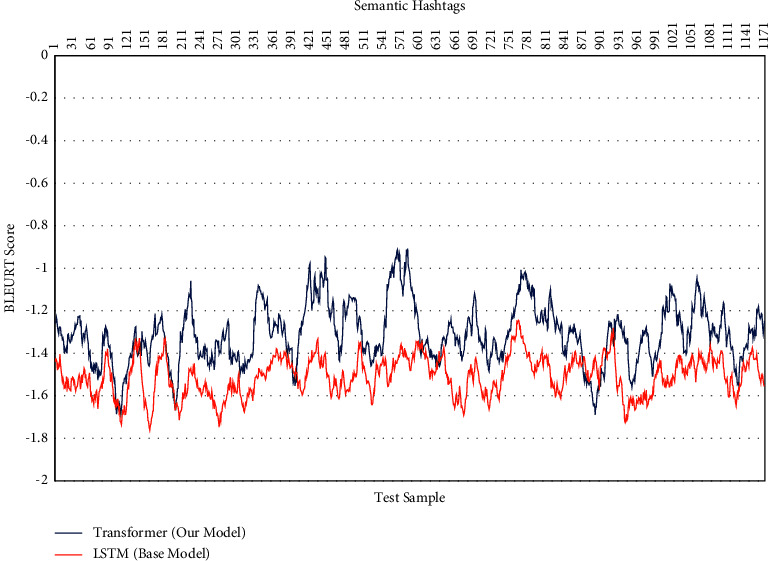
BLEURT score of semantic in generated hashtags (1200 test sample).

Figure 9 shows BLEURT score for each 1,200 images test sample. In Table 5, the average accuracy obtained for the 1,200 samples of images can be seen.

The samples of generated hashtags and hashtags entered by Instagram users are contrasted in the two tables above. The scores are lower than zero since the generated hashtags contain a word structure and because there is no grammatical structure. Even yet, a relatively good increase is visible in the sample of hashtags produced by the algorithm used in this study compared to the recurrent neural network approach.

## 6. Conclusion and Future Works

With the use of our model-generated hashtags, we hope to increase engagement for each post image by discovering a way to produce trending hashtags for each image. The experiment and evaluation section demonstrates a significant improvement over the base model (LSTM) in terms of producing popular hashtags that increase engagement for each post and better quality (semantically sound) hashtags. This work is just the start of creating well-known hashtags of this kind. The model given in this study can be used for various research projects, such as models for deep image recognition and condition-based element detection in images. It is also possible to propose further uses for this approach in the area of marketing and advertising.

Future work will produce more precise results for classifying image elements and a better result for producing trending hashtags by utilizing a better language model designed specifically for this type of work. These improvements will be made by employing different models and improving the code or dataset. We can investigate the volume of hashtags produced with other datasets in upcoming studies. The adoption of better hardware could also produce results that are superior to those of this investigation due to hardware restrictions. The popularity of hashtags was only conceptually examined in this study; a field investigation is needed. In other words, the research that shows how much use of these popular hashtags was generated increased views lead to a more accurate assessment of this model. Also, due to the day-to-day changes of popular hashtags, trying to provide a way to generate popular hashtags by recognizing the elements and objects of the image by day or hour is a practical idea for future research. In other words, the research that demonstrates how much use of these trending hashtags increased views contributes to a more realistic evaluation of this strategy. Additionally, trying to develop a method of generating popular hashtags by identifying the components and objects of the image by day or hour is a useful idea for future research due to the daily fluctuations of popular hashtags.

## Figures and Tables

**Table 1:** Statistics of the collected data.

Number of comments	4,572,833
Number of likes	115,588,219
Number of images	572,623
Number of hashtags	1,410,264
Number of unique hashtags	113,092
Number of users	105,089
Date of collected data	2010 to 2019

**Table 2:** BLEU evaluation based on 800 test data.

Base model (LSTM)	0.252
Popular hashtags with transformer	**0.344**

**Table 3:** BLEU evaluation based on 1200 test data.

Base model (LSTM)	0.262
Popular hashtags with transformer	**0.357**

**Table 4:** BLEURT evaluation based on 800 test data.

Base model (LSTM)	−1.528
Popular hashtags with transformer	**−1.302**

**Table 5:** BLEURT evaluation based on 1200 test data.

Base model (LSTM)	−1.509
Popular hashtags with transformer	**−1.301**

## Data Availability

The data used to support the findings of this study are available from the corresponding author upon request.
